# Pharmaceutically modified subtilisins withstand acidic conditions and effectively degrade gluten *in vivo*

**DOI:** 10.1038/s41598-019-43837-9

**Published:** 2019-05-16

**Authors:** Ghassan Darwish, Eva J. Helmerhorst, Detlef Schuppan, Frank G. Oppenheim, Guoxian Wei

**Affiliations:** 1 Department of Molecular and Cell Biology, Henry M. Goldman School of Dental Medicine 700 Albany Street, Boston, Massachusetts USA; 2 Division of Gastroenterology, Beth Israel Deaconess Medical Center, Harvard Medical School, Boston, Massachusetts USA; 3 Institute of Translational Immunology and Research Center for Immune Therapy, University Medical Center, Johannes-Gutenberg-University, Mainz, Germany

**Keywords:** Drug delivery, Coeliac disease

## Abstract

Detoxification of gluten immunogenic epitopes is a promising strategy for the treatment of celiac disease. Our previous studies have shown that these epitopes can be degraded *in vitro* by subtilisin enzymes derived from *Rothia mucilaginosa*, a natural microbial colonizer of the oral cavity. The challenge is that the enzyme is not optimally active under acidic conditions as encountered in the stomach. We therefore aimed to protect and maintain subtilisin-A enzyme activity by exploring two pharmaceutical modification techniques: PEGylation and Polylactic glycolic acid (PLGA) microencapsulation. PEGylation of subtilisin-A (Sub-A) was performed by attaching methoxypolyethylene glycol (mPEG, 5 kDa). The PEGylation protected subtilisin-A from autolysis at neutral pH. The PEGylated Sub-A (Sub-A-mPEG) was further encapsulated by PLGA. The microencapsulated Sub-A-mPEG-PLGA showed significantly increased protection against acid exposure *in vitro*. *In vivo*, gluten immunogenic epitopes were decreased by 60% in the stomach of mice fed with chow containing Sub-A-mPEG-PLGA (0.2 mg Sub-A/g chow) (n = 9) compared to 31.9% in mice fed with chow containing unmodified Sub-A (n = 9). These results show that the developed pharmaceutical modification can protect Sub-A from auto-digestion as well as from acid inactivation, thus rendering the enzyme more effective for applications *in vivo*.

## Introduction

Gluten proteins are found in wheat, barley and rye, and trigger celiac disease (CeD), a chronic inflammatory immune-mediated disease affecting the small intestine^1^. Immunogenic gluten proteins show unusual resistance to degradation by human digestive enzymes^2,3^. The surviving gluten-derived peptides reach the lamina propria in the duodenum where they are deamidated by the enzyme and CeD autoantigen tissue transglutaminase^4^. Immunogenicity is dependent on peptide length, and only peptides longer than 8–11 residues can induce an immune response. Therefore, detoxification of gluten can be achieved by their thorough proteolytic fragmentation. There are a few oral enzymes currently being explored to treat CeD, some of which have reached clinical trials^1,5^. Two enzymes, prolyl endopeptidase (PEP) from *Sphyngomonas capsulata* and a glutamine-specific cysteine endopeptidase (EP-B2) from barley, together being present in the formulation ALV003 (Alvine Pharmaceuticals), have been profoundly investigated for their effect on gluten inactivation^3,6–10^. A preliminary clinical study showed that the effectiveness of PEP from *F. meningosepticum* is restricted to the length of suitable substrate^11^, with optimal activity at neutral pH^12^.

A promising new source of gluten-degrading enzymes are the microbes that colonize the human digestive canal, starting in the oral cavity^13^. *Rothia* species are part of the commensal oral microflora^14^ and are generally considered harmless colonizers associated with oral health, although a few case reports of infections implicating *Rothia* have been described^15^. *Rothia mucilaginosa* as well as *Rothia aeria* exert a high gluten-degrading activity at pH 7.0. Both are capable to thoroughly cleave gluten after glutamine-proline-glutamine (QPQ) and leucine-proline-tyrosine (LPY) sequences which are abundant in immunogenic gluten domains^16–18^. The enzymes from *Rothia mucilaginosa* have been isolated in our laboratory and were identified as subtilisins^19^. The subtilisin enzymes, many being food-grade such as the ones from *Bacillus* species, could potentially benefit CeD patients if they could more effectively abolish gluten immunogenic epitopes during gastro-duodenal transit *in vivo*^19^.

Subtilisins are a group of serine proteases, with the *Bacillus* species showing a MW from 26 to 28 kDa. Subtilisin Carlsberg (subtilisin-A or Sub-A, 274 amino acids) is produced by *Bacillus licheniformis*^20^ and comprises two alpha-helices and a large beta-sheet structure. Given their food-grade status, subtilisins hold promise as digestive aides for gluten degradation, but similar to bacterial PEP their activity is dramatically reduced under acidic conditions, as prevail in the stomach.

Stability and therapeutic potency of proteins can be improved through the application of pharmaceutical modification and/or enteric coating techniques that permit the drug’s release in the target area of interest, e.g., the small intestine^21,22^.

The PEGylation technique consists of covalent and non-covalent attachment of polyethylene glycol (PEG) a varying chain length to a molecule such as a therapeutic protein, thereby enhancing its protection from proteolytic degradation and increasing its stability^23,24^. Usually, PEG is attached to the ***ɛ***-amine group in lysine residues^25^. PEGylated proteins can be further modified by enteric coating techniques, e.g. microencapsulation, to achieve further protection in the digestive canal.

PLGA is an FDA approved food-grade polymer. Its features such as being water-insoluble, predictability of degradation, ease of fabrication, strength, hydrophobicity, biocompatibility and pliability make PLGA a preferred polymeric vehicle for medical applications^26–28^.

To address the issue of acid sensitivity and autolysis of subtilisin enzymes under acidic conditions, we modified Sub-A, which showed similar activity as the *Rothia* subtilisins^19^ and was readily commericially available, with FDA-approved pharmaceutical conjugation (PEGylation) and microencapsulation (emulsion) techniques. Activity analysis *in vitro* and *in vivo* of the modified enzymes showed a significant protection from inactivation by gastric acid, while increasing activity in the small intestine. This led to a significantly improved elimination of immunogenic gluten epitopes *in vivo*. Since this technology is generally applicable, it should be useful not only for Sub-A enzymes but also for other glutenases considered for clinical application that are acid sensitive and/or prone to autodegradation.

## Results

### Sub-A undergoes autolysis at neutral pH

**Figure 1:**
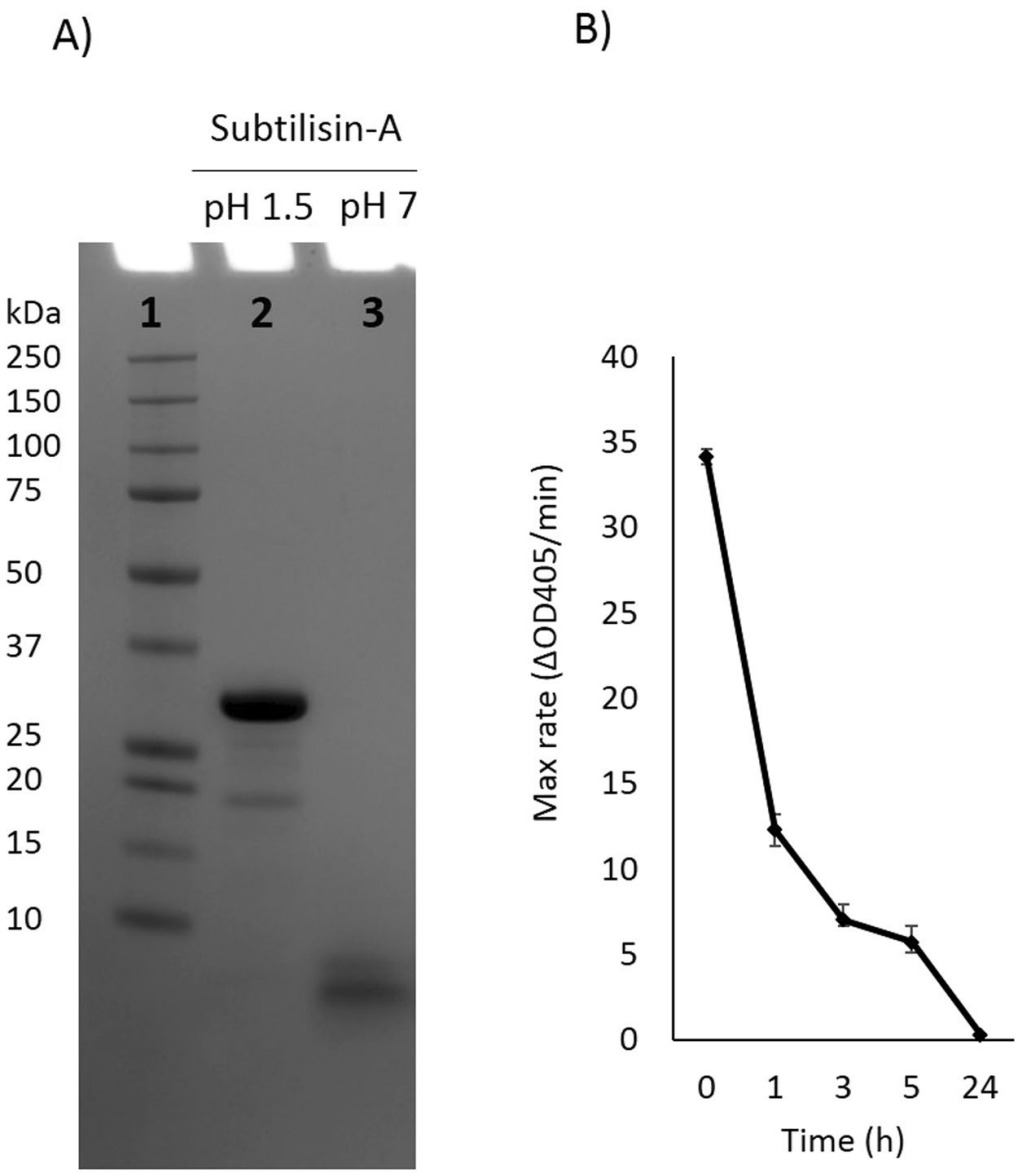
Sub-A protein composition and activity. (**A**) Protein composition of the enzyme dissolved at pH 1.5, and at pH 7.0, analyzed by SDS-PAGE. Lane 1: MW; Lane 2: Sub-A in pH 1.5; Lane 3: Sub-A in pH 7.0; (**B**) Enzyme activity over time, analyzed using Suc-AAPF-pNA as the enzyme substrate.

When Sub-A (MW 27 kDa) was dissolved at neutral pH, a band with a much lower molecular weight (<10 kDa) was observed within 10 min (Fig. 1A). The enzymatic activity of subtilisin incubated at 37 °C decreased over time, and this loss of activity must be attributed to autolysis (Fig. 1B). The results suggested that Sub-A undergoes autolysis at neutral pH as prevails in the small intestine.

### Sub-A is inactive at low pH

**Figure 2:**
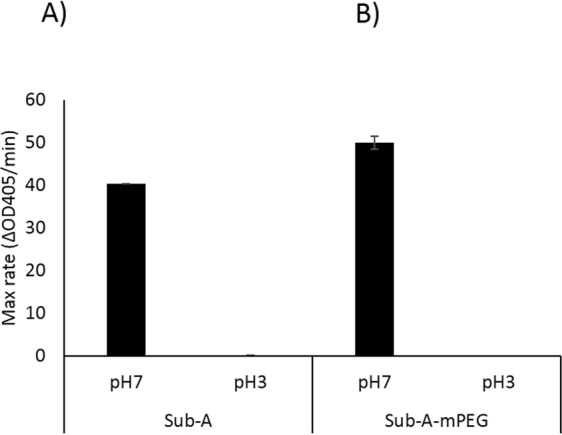
Activity of Sub-A and PEGylated Sub-A (Sub-A-mPEG). The activity was analyzed at pH 7.0 and at pH 3.0, n = 3. (**A**) Sub-A; (**B**) Sub-A-mPEG.

Sub-A was highly active in hydrolyzing the Suc-AAPF-pNA substrate at neutral pH (intestinal buffer, pH 7.0) (Fig. 2A). However, the enzyme was inactive at low pH (gastric buffer, pH 1.5–3.0). Even short-term (10 min) exposure of Sub-A to low pH inactivated the enzyme, since activity could not be recovered upon transfer to neutral pH (Fig. 2A). Both autolysis (Fig. 1) and inactivation at low pH are a challenge for application of gluten degrading enzymes in the digestive tract. Therefore, pharmaceutical modification was pursued to render the enzyme more resilient.

### PEGylation of Sub-A

Sub-A was modified by PEGylation with mPEG (Sub-A-mPEG). As shown in Supplemental Fig. 1 and Supplemental Table 1, the extent of mPEG modification in Sub-A-mPEG was 81.6% (~8 of 10 primary amines being PEGylated) as determined by the fluorescamine method (A) and 55.4% (~6 of 10 primary amines being PEGylated) as determined by the 2,4,6-Trinitrobenzenesulfonic acid (TNBSA) method (B). The yield of Sub-A-mPEG, assessed by the weight of the modified product (Sub-A-mPEG)/total weight of the agents before modification (Sub-A + mPEG) × 100%) was ~73%, and the PEGylated products contained ~34% (w/w) Sub-A (Supplemental Table 2).

### PEGylated subtilisin-A (Sub-A-mPEG) activity

**Figure 3:**
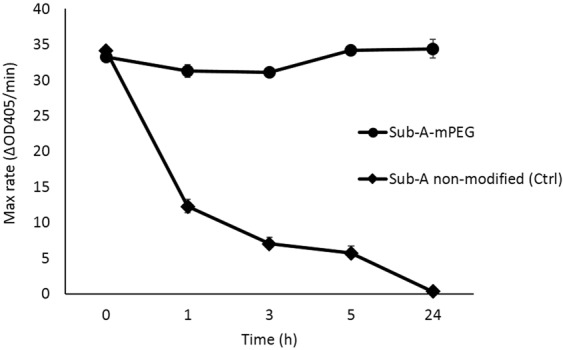
PEGylation protects Sub-A from autolysis. The activity of Sub-A and Sub-A-mPEG was monitored over time at pH 7.0, n = 3.

PEGylation of Sub-A had a dramatic effect on its autolytic activity (Fig. 3). While Sub-A lost activity over time, the partial modification of the free amines by PEGylation virtually completely prevented autolysis. This can be explained by steric hindrance and partial modification of the free N-termini in the enzyme structure, which could impede autodegradation by impeding Sub-A domains to fold towards the active site. The slightly higher average activity of PEGylated Sub-A compared to unmodified Sub-A was not statistically significant.

### PLGA encapsulation of Sub-A-mPEG

While autolysis could be effectively prevented with mPEG modification, the fact remained that Sub-A mPEG is irreversibly inactivated at low pH, as shown in Fig. 2B. Therefore, to obtain an enzyme preparation that could withstand gastroduodenal transit, further pharmaceutical modification of Sub-A-mPEG was needed.

To protect Sub-A-mPEG from acidic insults, the enzyme was coated with PLGA. The yield of Sub-A-mPEG-PLGA, as determined by the weight of the modified product (Sub-A-mPEG-PLGA)/total weight of the agents before modification (Sub-A-mPEG + PLGA) × 100%, was ~43%. The Sub-A concentration in Sub-A-mPEG-PLGA was ~8% (w/w) as shown in Supplemental Table 2.

**Figure 4:**
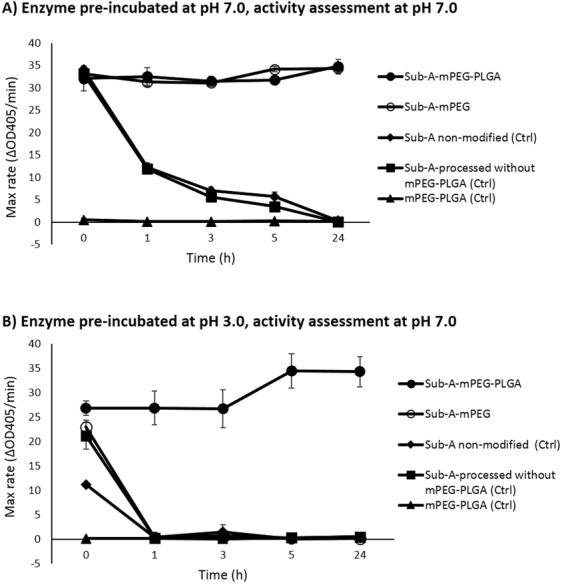
mPEG-PLGA modification renders Sub-A resistant to acid exposure. (**A**) Enzyme preparations pre-incubated at pH 7.0 and tested at pH 7.0; (**B**) Enzyme preparations pre-incubated at pH 3.0 and tested at pH 7.0, n = 3.

Significant improvements with respect to acid resistance of the enzyme were achieved by PLGA microencapsulation. In Fig. 4A, the activity of all compounds, incubated at pH 7.0 and subsequently tested at pH 7.0 are compared. Sub-A processed without the addition of PLGA or mPEG (unmodified control) was active, but the activity decreased over time due to autolysis. This autolysis was prevented by mPEG modification, as evidenced by the sustained high activity of Sub-A-mPEG over time. PLGA-encapsulated Sub-A-mPEG (Sub-A-mPEG-PLGA) was also active, and retained activity over time. This is expected since after dissolution of the PLGA coating at neutral pH the active Sub-A-mPEG enzyme is released.

Next, the effect of PLGA coating on acid resistance was investigated. The compounds were pre-incubated in low pH buffer (pH 3.0), and then transferred to a buffer with a neutral pH. The Sub-A controls, as well as Sub-A-mPEG were inactive after the acid challenge. However, Sub-A-mPEG-PLGA was fully active after the acid challenge (Fig. 4B). These results indicate that the PLGA coating had protected the encapsulated Sub-A-mPEG enzyme from denaturation during the acid exposure.

### Gliadin hydrolysis by Sub-A-mPEG-PLGA

**Figure 5:**
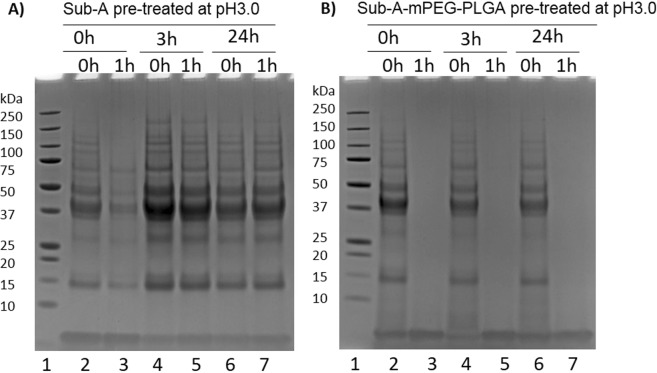
Sub-A-mPEG-PLGA after acid challenge can degrade gliadins. Sub-A (**A**) or Sub-A-mPEG-PLGA (**B**) were incubated at 37 °C in acidic conditions (pH 3.0) for 0, 3, or 24 hr, and then transferred to pH 7.0. Enzyme activity was monitored at t = 0 h and t = 1 h using mixed gliadins as the substrate. Lane 1: MW; Lane 2, 4, 6: Sub-A or Sub-A-mPEG-PLGA in pH 3.0 for 0, 3, 24 hr, then gliadin added and incubated for 0 hr; Lane 3, 5, 7: Sub-A or Sub-A-mPEG-PLGA in pH 3.0 for 0, 3, 24 hr, then gliadin added and incubated for 1 hr.

To assess the activity of Sub-A and Sub-A-mPEG-PLGA towards a gluten substrate, both enzyme preparations were exposed to acidic conditions at 37 °C for 0, 3 and 24 hr, and then incubated with mixed gliadins (the major fraction of gluten proteins) at neutral pH and at 37 °C for 0 or 1 hr. Gliadin degradation was monitored by SDS-PAGE. Figure 5A shows the results obtained with Sub-A, and Fig. 5B the results obtained with Sub-A-mPEG-PLGA. After acid exposure, Sub-A was inactive, as evidenced by the fact that gliadins remained intact after mixing with the enzyme. However, Sub-A-mPEG-PLGA induced gliadin degradation after acid exposure for 24 hr, which indicates the enzyme remained active even long after acid challenge (Fig. 5B, lane 3, 5 and 7). These results confirm that Sub-A-mPEG-PLGA can withstand acid challenge and that its Sub-A-mPEG component can efficiently degrade gliadins once released, without significant autolytic degradation.

### Detoxification of gliadin immunogenic epitopes *in vivo*

**Figure 6:**
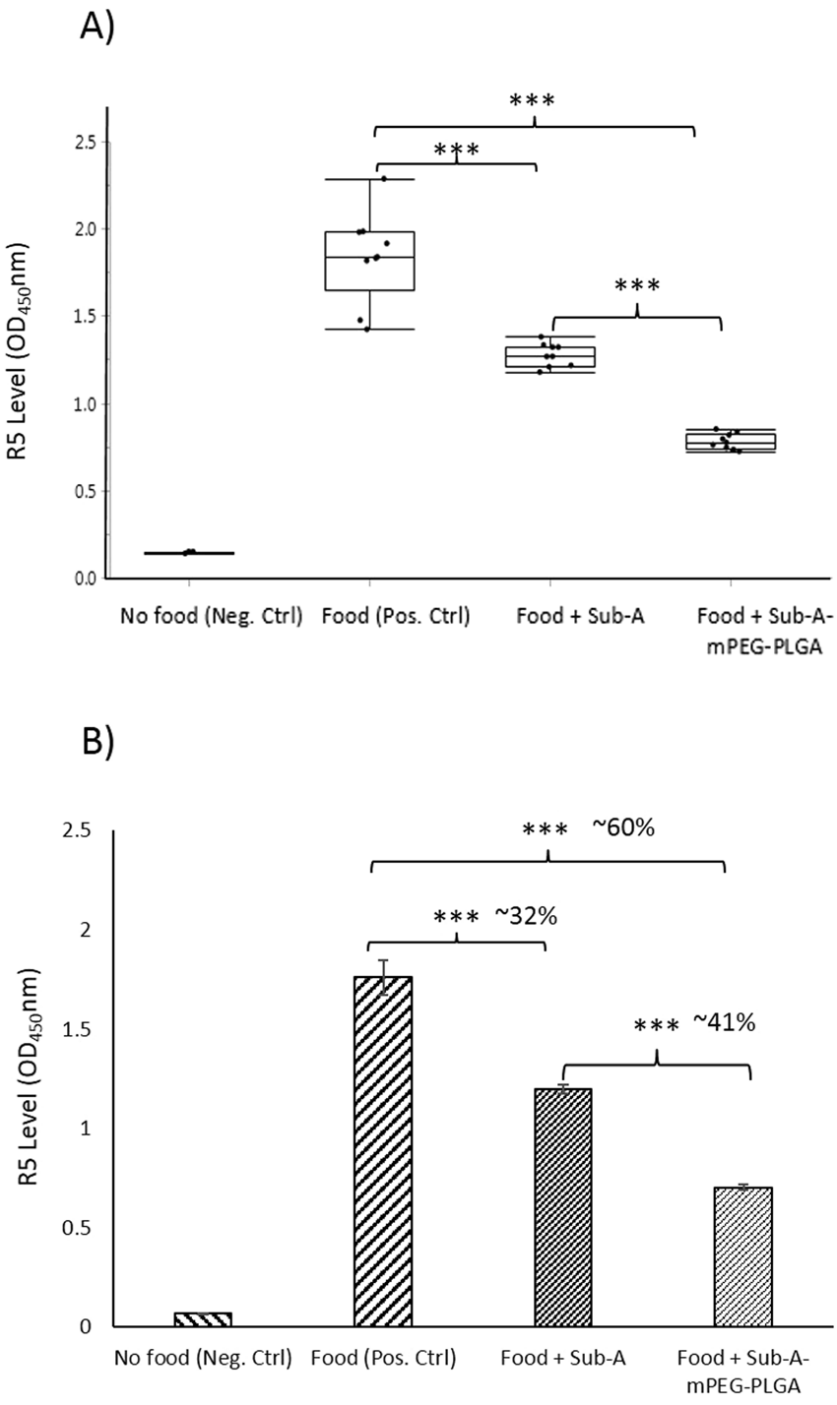
Sub-A-mPEG-PLGA is active *in vivo*. Three groups of mice were treated either with gluten-containing chow only (n = 9), chow supplemented with Sub-A (n = 9) or chow supplemented with Sub-A-mPEG-PLGA. After 3 hr, mice were euthanized, stomach contents harvested, and the survival of the R5 epitopes was determined via ELISA. (**A**) Box plot showing the individual data points; (**B**) Average +/− SE. Data were analyzed with SPSS. ***p < 0.001.

To experimentally explore if Sub-A-mPEG-PLGA would be able to degrade and abolish immunogenic gliadin epitopes during gastro-intestinal transit, an *in vivo* experiment was carried out. Three groups of 9 mice each were fasted for 18 hr, and received either gluten containing chow alone, gluten-chow supplemented with Sub-A or with Sub-A-mPEG-PLGA. The mice ingested ~1 g chow in 1 hr. After digestion for two more hours, the stomach contents of the mice were harvested, gliadin peptides extracted and quantified for the R5 epitope, which represents a highly immunogenic gliadin peptide domain that is present in many gliadin molecules using The R5-ELISA. As shown in Fig. 6, gluten immunogenic epitopes in the stomach of the mice fed gluten-chow and non-modified Sub-A were reduced by 31.9%. Notably, the reduction of R5-epitopes was significantly higher 60.0%, (*p* < 0.01) in mice fed with gluten-chow supplemented with Sub-A-mPEG-PLGA. This showed that the efficacy of gliadin degradation *in vivo* was doubled after enzyme modification with mPEG and encapsulation by PLGA. Overall, Sub-A in this twofold modified form more efficiently removes immunogenic epitopes from the gastric lumen, which may facilitate to digest epitopes in the pH-neutral duodenal region.

## Discussion

Protein-based drugs hold promise as therapeutic agents because of their high specificity, but they often display short half-life’s *in vivo*, either due to rapid excretion or proteolysis. Instability and autodegradation of enzymes are major challenges for their therapeutic application. PEG modification usually improves protein stability^29^ and encapsulation of chymotrypsin in PLGA microspheres prevented loss of its enzymatic activity^29^. Here we combined PEGylation and PLGA microencapsulation to protect the gluten degrading enzyme subtilisin-A from inactivation due to acid exposure as well as autolysis at neutral pH.

Subtilisins have a potential to be used for therapeutic application in the treatment of CeD due to their gluten-degrading activity and their food grade availability. However, subtilisin-A, like many other enzymes, is only weakly active under acidic conditions^30^. Sub-A activity was lost at low pH, and could not be recovered after transfer to neutral pH, suggesting that the enzyme’s active site is denatured at low pH. PLGA microencapsulation efficiently stabilized the enzyme under acidic conditions.

PEGylation successfully protected Sub-A autolytic degradation, while retaining its ability to digest externally added substrates. The size of mPEG has been reported as a factor to impact the efficiency of PEG modification. mPEG with molecular weights ~5 kDa have been found to be most efficient for protecting enzyme proteins^29,31,32^. Larger MW (>10 kDa) PEG molecules tend to fold and occupy a large surface area of the protein, interfering with substrate binding ability^33,34^. Our modification using 5 kDa mPEG yielded a high conjugation efficiency (~55%), which is comparable to studies with other protein substrates in which the PEG modification efficiency ranged between 50–60%^32,35^.

We investigated the extent of mPEG modification by the fluorescamine and the TNBSA assays^31^. TNBSA recognizes the primary amines in unfolded proteins, compared to the fluorescamine that detects amines in folded proteins. Sub-A has 10 primary amines including 9 Lysine (K) residues and one free N-terminus^36^. We showed that 8 vs 6 of the 10 primary amines in Sub-A were occupied as determined by fluorescamine vs TNBSA in line with 2 internal amines that are inaccessible in the native stet of the enzyme and 6 PEGylated primary amines. Importantly, PEGylation of these 6 amines did not compromise enzymatic activity of Sub-A, as shown in Fig. 4. ~60% PEGylation has been demonstrated previously to induce no significant changes in secondary and tertiary structure of the enzyme^32^, retaining 87% activity^37^. It is possible that a lower degree of PEGylation may improve enzyme activity, while not dramatically compromising its stability, but such optimization will require further studies.

Our *in vitro* and *in vivo* studies clearly demonstrate that PLGA microencapsulation (Sub-A-mPEG-PLGA) dramatically protected the enzyme against acid denaturation. The mechanisms of drug release from PLGA coatings include a collective PLGA degradation process of bulk or surface diffusion and erosion neutral vs acidic pH^38,39^. In addition, PLGA degradation depends on many factors such as molecular weight of the polymer, the degree of crystallinity, the glass-transition temperature of the polymer, and the size and shape of the matrix^40^.

The enzyme type, load, and conjugation also impact the release rate and pattern of the coated enzyme from PLGA^40^. The presence of the PEG polymer conjugated with the enzyme likely affects the release profile of Sub-A. Since there are many variables that influence the degradation process, the release rate and patterns are often unpredictable, especially *in vivo* where the pH conditions can fluctuate in time, depending on the ingested food and depending on the anatomical location.

The animal experiment confirmed the *in vitro* finding, where the encapsulated enzyme was better protected than subtilisin-A and twice as efficient in degrading gliadins. Activity was only measured in the stomach contents. It can be envisions but needs to be shown that activities would even be higher in the neutral duodenal environment, where PLGA dissolution and enzyme release are be favored.

Overall, the combination of mPEG and PLGA coating of subtilisins provides a novel strategy to deliver otherwise acid-sensitive gluten-degrading enzymes to the gastrointestinal tract with enhanced activity and stability. To our knowledge, this is the first study to demonstrate a synergistic effect of PEGylation and PLGA encapsulation for enteric coating to preserve the activity as well as stability of food grade subtilisins. The combination of PEGylation and microencapsulation could be used to modify other gluten-degrading enzymes, including, e.g., prolyl endopeptidases, and serve as a general tool for a more efficient glutenase therapy of celiac disease.

## Materials and Methods

### Modification of Sub-A

**Figure 7:**
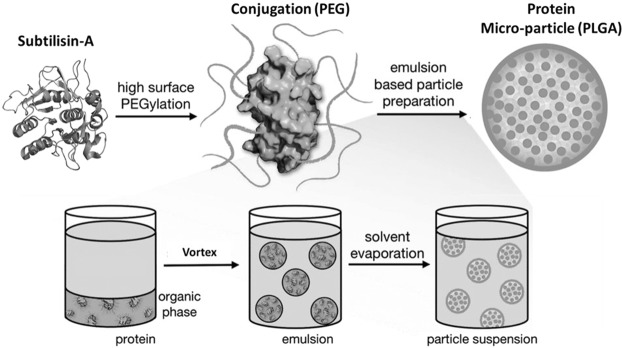
Schematic representation of the pharmaceutical coating procedure applied to Sub-A. [Radi *et al*., Med. Chem. Commun. 2016, 7, 1738–1744] - Reproduced by permission of The Royal Society of Chemistry. All rights reserved.

Sub-A from *B. licheniformis* (Sigma, product number 5380) was modified by PEGylation and microencapsulation as schematically representated in Fig. 7.

### PEGylation of Sub-A

PEGylation of Sub-A was performed using the method reported by Castellanos^41^ and Mabrouk^42^. Briefly, Sub-A (40 mg) and activated mPEG (120 mg, 5 kDa) were dissolved in 20 ml of 0.1 M sodium borate buffer (pH 9.2) at an approximate molar ratio of 1:3 (solvent-accessible lysine residues in Sub-A to mPEG) and stirred for 3 hr at 4 °C. The reaction was quenched by the addition of 20 ml of 0.1 M potassium phosphate buffer (pH 7.0). Non-reacted mPEG and buffer salts were removed by dialysis using membranes with a cut-off of 8 kDa (Spectra Medical Industries, Laguna Hills, CA) against 1 L of miliQ water for 24 hr. The PEGylated Sub-A (Sub-A-mPEG conjugates) in aqueous solution was frozen in −80 °C for 30 min and lyophilized (Virtis FM 25 EL, NY, USA) at a condenser temperature of −70 °C and a pressure of <20 millitorr. Lyophilized protein powders were kept at −20 °C until used in the experiments.

### Validating PEGylation efficiency

The PEGylation efficiency of Sub-A was evaluated with the fluorescamine method for folded proteins^31,43,44^ and the TNBSA methods for unfolded proteins^31^.

#### Fluorescamine Assay

The average number of free amino groups in the PEGylated Sub-A surface was determined by a fluorescamine method^31,43,44^. Fluorescamine, a heterocyclic dione, reacts with primary amines to form a fluorescent product. The fluorescence of a solution containing protein plus fluorescamine is proportional to the quantity of free amine groups present. Unmodified and mPEG-modified Sub-A were dissolved in PBS (pH 7.4) at 0, 0.1, 0.2 and 0.3 mg/ml, and aliquots (150 μL) was pipetted into each well of a 96-black-well microplate (flat bottom) in triplicate. The fluorescence was measured immediately after mixing with 50 μL of 0.3 mg/mL fluorescamine solution (in acetone) at an excitation wavelength of 360 nm, and an emission wavelength of 465 nm. Fluorescence intensity values were plotted versus the Sub-A concentrations. The percentage of mPEG modification was calculated using the formula [1 − (slope of Sub-A-mPEG/Sub-A)] × 100.

#### TNBSA Assay

Since fluorescamine cannot react with unexposed primary amines of folded proteins, the TNBSA (2,4,6-Trinitrobenzenesulfonic acid) method^45^ was used to further estimate the extent of mPEG modification. The protein is unfolded in HCl and SDS, rendering all amino groups solvent accessible. The assay was performed as follows: Sub-A-mPEG and Sub-A were dissolved in 1 ml of 0.2 M sodium bicarbonate buffer (pH 8.5) to achieve concentrations between 0.1 and 0.4 mg/ml. To these solutions and buffer blanks, 0.25 ml of 0.01% TNBSA (w/v), 0.25 ml of 10% SDS solution (w/v), and 0.125 ml of 1 N HCl were added. The mixtures were incubated at 37 °C for 2 hr, and their absorbance at 335 nm was subsequently determined (Genios microtiter plate reader, Tecan, Männedorf, Switzerland). The absorbance values were calibrated by subtracting the values of buffer blanks and were then plotted versus the Sub-A/Sub-A-mPEG concentration. The percentage of mPEG modification was calculated using the formula [1 − (slope of Sub-A-mPEG/Sub-A)] × 100.

### Microencapsulation of PEGylated Sub-A

Microencapsulation of PEGylated Sub-A was performed essentially as described^29^. Sub-A-mPEG (10 mg) was dissolved in 0.5 ml of 0.2 M sodium bicarbonate buffer (NaHCO3, pH 10.0), and poly-lactic glycolic acid (PLGA) (90 mg) was dissolved in 0.5 ml acetone. The two solutions were mixed, vortexed, followed by addition of 3 ml of light liquid paraffin and 0.05 ml of Span 80 while with vortexing. The mixture was placed in 40 ml beaker while stirring at 500–700 rpm using for 3 hr at room temperature until the acetone evaporated completely, resulting in the formation of microcapsules. The formed microcapsules were harvested by centrifugation (2 min, 7,000 rpm), washed (5x) in petroleum ether (40–60° grade), and dried at room temperature for 24 hr.

### Acid challenge test

To test the stability and activity of Sub-A and Sub-A-mPEG after incubation under acidic conditions, the enzymes were dissolved in diluted gastric buffer (pH 3.0, Sigma 01651) at a concentration of 0.25 mg/ml. After incubation at 37 °C for 1 hr, the solutions were neutralized and diluted by adding an intestinal buffer (pH 7.0, Sigma 53757). The enzymes (0.25 µg/ml) were then tested for activity using Suc-AAPF-pNA as the substrate.

### Enzyme activity assessment using p-nitroanilide-derivatized peptide

The aliquots (50 µl) of Sub-A, Sub-A-mPEG, or Sub-A-mPEG-PLGA were added to 150 µl of PBS (pH 7.0) and diluted to 0.25 µg/ml. The 200 µl aliquots were added in triplicate to a 96-well microtiter plate (Costar 3596, Corning, NY, USA) mixed with 0.2 mM n-succinyl-ala-ala-pro-phe-p-nitroanilide (Suc-AAPF-pNA, Sigma). Substrate hydrolysis was spectrophotometrically monitored at 405 nm, at 37 °C for 1 hr at 10 min intervals using a Genios microtiter plate reader (Tecan, Männedorf, Switzerland).

### Enzyme activity assessment using gliadins

Sub-A and Sub-A-mPEG-PLGA were dissolved in diluted HCl buffer (pH 3.0) to 1 mg/ml and incubated at 37 °C for 0, 3 and 24 hr. The 24 hr time point was chosen to capture any low enzyme activity that might be present in the sample. Gliadin (Sigma) hydrolysis activity of each pre-treated enzyme sample were determined by incubating a 40 fold dilution of the pretreated enzyme (25 µg/ml) samples with gliadin (250 µg/ml) in PBS (pH 7.0) at 37 °C. Gliadin degradation was monitored after 0 and 1 hr of incubation in 100 µl sample aliquots subjected to 4–12% SDS PAGE.

### Assessment of enzyme activity *in vivo*

**Figure 8:**
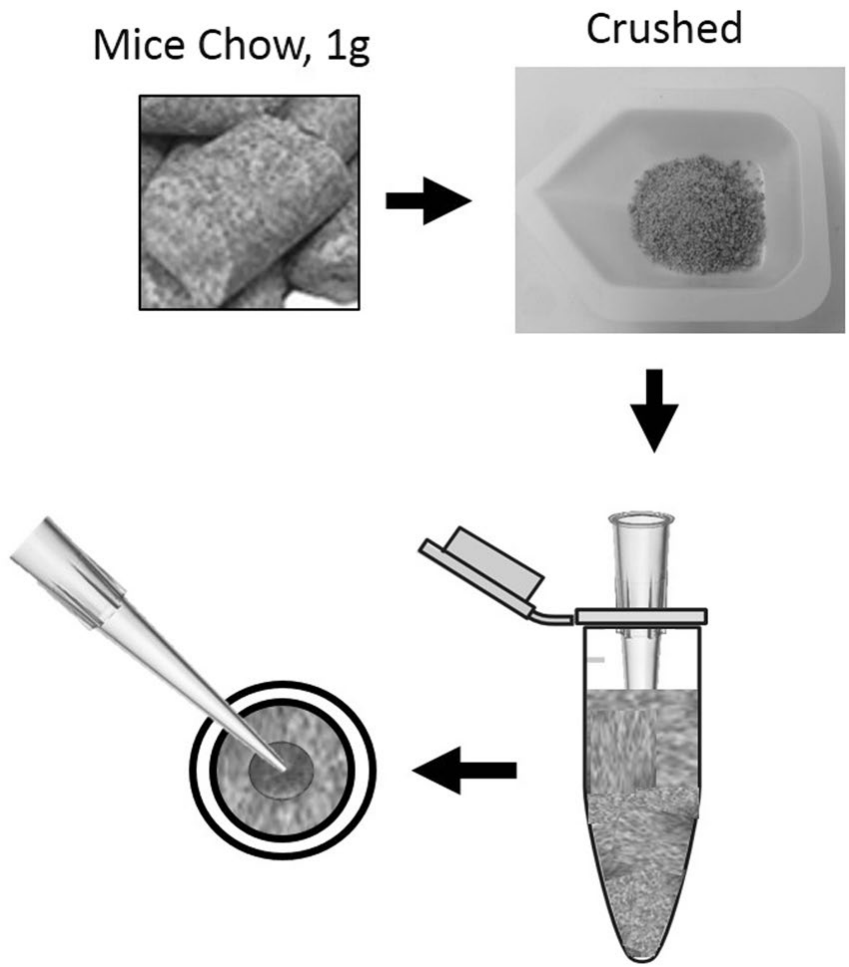
Method for the preparation of mouse chow with added enzyme (Sub-A or Sub-A-mPEG).

All animal experiments were conducted using institutionally approved protocols by Boston University’s Institutional Animal Care and Use Committee (IACUC) and Animal Research: Reporting of *In Vivo* Experiments (ARRIVE) guidelines. The animals were housed in a controlled environment (22 °C, 12 hr day/night cycle) with ad libitum food and drink access. For the *in vivo* experiment, 1 g of powdered mouse chow (2018, Envigo, Cambridgeshire, UK), containing 18% crude proteins where ~50 mg gluten or ~25 mg gliadin/g chow included, was added into a 2 ml Eppendorf tube and mixed incrementally with 400 to 600 µl sterilized water by packing layer by layer to form a pellet, where a 200 µl-yellow pipette tip was inserted to cast a hollow center for loading of the enzyme samples (Fig. 8). The chow pellets were dried in the SpeedVac machine (SpeedVac plus SC110 A, Savant, NY, USA) for 3 hr. Sub-A or Sub-A-mPEG-PLGA were added in an amount of 0.2 mg to the center of the pellets, which were then closed with the mice chow paste.

Balb/c female mice (9 weeks old, n = 30) were acclimatized for a week. After 18 hr of fasting, the mice were fed for 3 hr as follows: Group I (n = 9): chow pellets without added enzyme; Group II (n = 9): chow pellets with Sub-A; and Group III (n = 9): chow pellets with Sub-A-mPEG-PGLA; and no food controls (n = 3). The mice were then euthanized, and stomach contents were collected and suspended in 2 ml of sterile water, and boiled (100 °C, 10 min), and then centrifuged (2,000 g, 2 min, at 4 °C). The suspensions were collected then mixed with 60% ethanol to extract gliadin peptides. After shaking (40 rpm) at room temperature for 1 hr, the mixtures were centrifuged (2,000 g × 10 min, 4 °C), and the ethanol extracted sample supernatants were collected. The protein content of the supernatants were determined by using the BCA protein assay kit (Sigma).

The concentration of immunogenic gluten epitopes of the extracted samples were subsequently analyzed using the ELISA-R5 assay kit (RIDASCREEN Gliadin, R-Biopharm, Darmstadt, Germany). This test measures the QQPFP, QQQFP and LQPFP epitopes sequence that occurs as important T cell stimulatory peptide epitope in several gliadin molecules^17^. The antibody coated microtiter plate wells were blocked with 1% defatted milk powder in PBS (200 µl). After incubation at room temperature for 1 hr, the plate was washed for three times with PBST (PBS + 0.5% Tween-20). The protein concentration of the stomach samples were adjusted to 160 µg/ml, and diluted 3,200-fold in dilution buffer. This dilution factor was determined to reduce gliadin levels to concentrations falling within the linear part of the standard curve. Aliquots of 100 µl of each of the diluted samples, as well as the controls of 0 ppb and 80 ppb gliadin standard solutions included in the kit, were added to each well, followed the instructions provided by the manufacturer.

### Statistics

Data were analysed using SPSS 17.0 software and were computed with GraphPad Prism 8. Standard one way ANOVA (non-parametric, when samples failed normality or equality of variance statistical tests) were used to test for statistical significance between groups. The data were represented as average ± standard error of mean (SEM). A value of *p* < 0.05 was considered to be statistically significant.

## Supplementary information

Supplementary information
