# Supplementary material for: Pharmaceutically modified subtilisins withstand acidic conditions and effectively degrade gluten in vivo
Source: Sci Rep. 2019 May 16;9:7505. doi: 10.1038/s41598-019-43837-9 (PMC6522598; doi:10.1038/s41598-019-43837-9)
Supplement: Supplementary file 1 — Supplementary information [file 41598_2019_43837_MOESM1_ESM.pdf]

Supplementary information

Pharmaceutically modified subtilisins withstand acidic conditions and effectively degrade gluten *in vivo*

Ghassan Darwish[1], Eva J. Helmerhorst[1], Detlef Schuppan[2,3], Frank G. Oppenheim[1], Guoxian Wei [1*]

[1] Department of Molecular and Cell Biology, Henry M. Goldman School of Dental Medicine 700 Albany Street, Boston, Massachusetts

[2] Division of Gastroenterology, Beth Israel Deaconess Medical Center, Harvard Medical School, Boston, Massachusetts; and

[3] Institute of Translational Immunology and Research Center for Immune Therapy, University Medical Center, Johannes-Gutenberg-University, Mainz, Germany

* Corresponding author:

Guoxian Wei, M.S., Ph.D.

Dept. of Molecular and Cell Biology

Boston University, Henry M. Goldman School of Dental Medicine

700 Albany Street, CABR W202B

Boston, MA 02118

Phone: 617-638-4916

Fax: 617-638-4924

Email: weigx@bu.edu

Supplementary information

(A) Fluorescamine

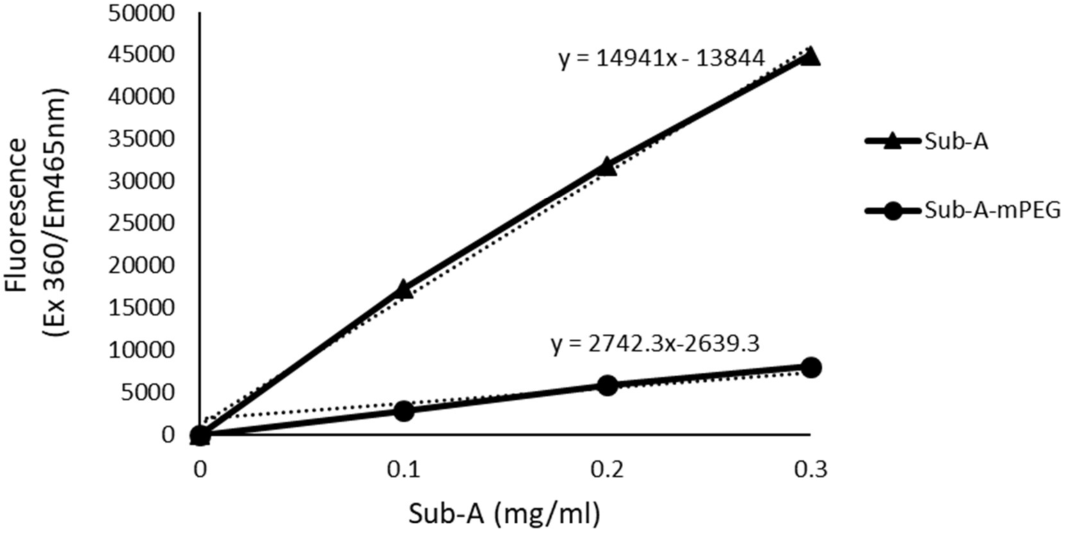

(B) TNBSA

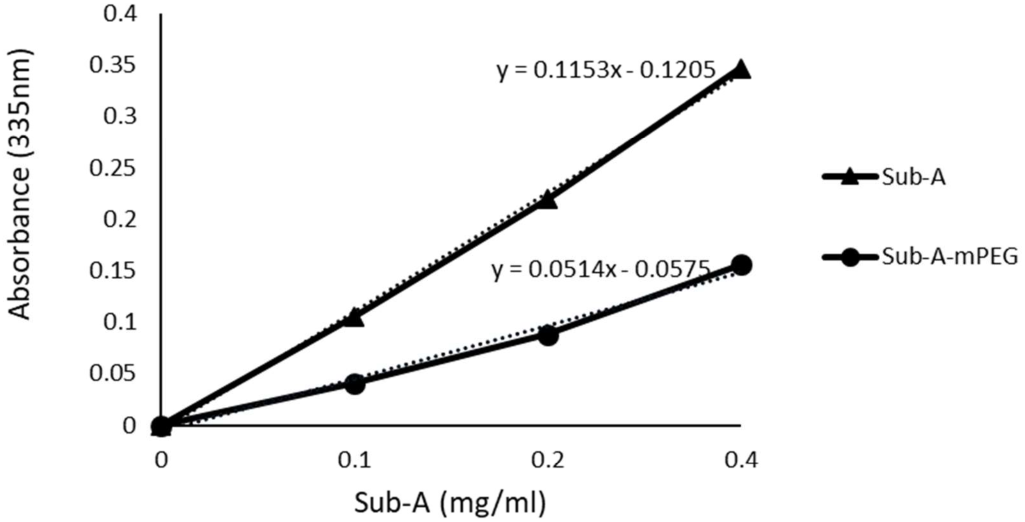

Supplemental Figure 1. Assessment of the extent of Sub-A PEGylation by (A) fluorescamine methods and (B) the TNBSA method, n=3.

Supplemental Table 1. Validation of PEGylation of Subtilisin-A (Sub-A) with Two Methods

**Methods**	**(A) Fluorescamine**	**(B) TNBS**
Target	Primary amine in folded protein	Primary amine in unfolded protein
Measurement	Fluorescent compound	Chromogenic product
Slope of Sub-A	14941	0.1153
Slope of Sub-A-mPEG	2742.3	0.0514
mPEG modification (%)	81.64	55.42
*PEGylated amines	~8	~6

*Number of primary amines in Sub-A is 10.

The data shown are representative of experiments conducted in triplicate.

Supplemental Table 2. Modification of Subtilisin-A (Sub-A)

	Before modification (mg)	After modification (mg)	Yield (%)	Sub-A Conc. (%, w/w)
Sub-A + mPEG	40+120=160	116.6	~73	~34
Sub-A-mPEG + PLGA	10+90=100	43.3	~43	~8

The data shown are representative of experiments conducted in triplicate.
